# Purpura anularis teleangiectodes: Fallbericht und Literaturübersicht

**DOI:** 10.1007/s00105-020-04667-3

**Published:** 2020-08-15

**Authors:** Jana Burghaus, Alexander Enk, Ferdinand Toberer

**Affiliations:** Abteilung Dermatologie, Venerologie und Allergologie, Universitätsklinikum, Ruprecht-Karls-Universität Heidelberg, Im Neuenheimer Feld 440, 69120 Heidelberg, Deutschland

**Keywords:** Purpura Majocchi, Petechien, Teleangiektasien, Chronisch venöse Insuffizienz, Hämorrhagisch pigmentierte Dermatose, Majocchi’s disease, Petechiae, Telangiectasias, Venous stasis, Pigmented purpuric eruptions

## Abstract

Die Purpura anularis teleangiectodes (PAT) ist eine seltene Erkrankung aus dem Spektrum der hämorrhagisch pigmentierten Dermatosen, die vorzugsweise junge Frauen betrifft und klinisch durch symmetrische, anuläre Erytheme mit Teleangiektasien an der unteren Extremität imponiert. Histologisch zeigen sich oberflächlich dermal gelegene Erythrozytenextravasate begleitet von einem lymphozytären Entzündungsinfiltrat. Als Auslöser können häufig Medikamente identifiziert werden. In idiopathischen Fällen werden kortisonhaltige Externa eingesetzt. Auch eine Kompressionsbehandlung kann unterstützend sinnvoll sein.

## Abstract (en)

Pupura annularis telangiectodes (PAT) is a rare entity belonging to the spectrum of the pigmented purpuric dermatoses. PAT presents clinically as symmetric, annular erythema with teleangiectasia on the lower extremities and preferably affects young women. Histology usually reveals extravasated erythrocytes accompanied by a lymphocyte-dominated inflammatory infiltrate in the superficial dermis. Medication can often be identified as causative. In patients with idiopathic disease, topical corticoidsteroids are the treatment of choice. Compression therapy may be supportive.

## Anamnese

Eine 39 Jahre alte Patientin stellte sich mit seit 4 Wochen größenprogredienten Hautveränderungen an beiden Unterschenkeln vor. Erstmalig habe sie die Läsionen vor 2 Monaten bemerkt. Es war kein Juckreiz vorhanden. Die Patientin verneinte kürzlich stattgehabte Infekte und Fieber. Auch ein Zeckenbiss sei nicht erinnerlich. Vorerkrankungen oder eine Dauermedikation bestünden nicht, auch seien vor Auftreten der Hautläsionen keine neuen Medikamente eingenommen worden. Seit 2 Wochen werde nun bei positiver Borrelienserologie (Ig[Immunglobulin]M) Doxycyclin eingenommen. Zu einer Besserung der Hautveränderungen habe diese Therapie jedoch nicht geführt.

## Befund

**Figure 1:**
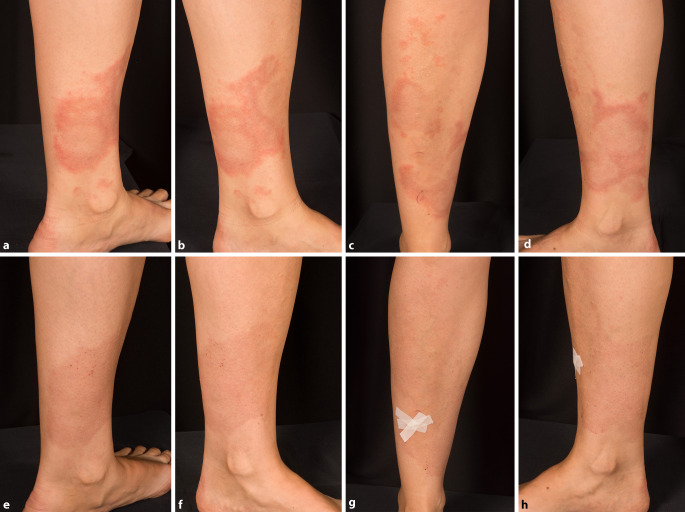

Klinisch zeigten sich an beiden Unterschenkeln nicht erhabene, anulär konfigurierte Erytheme mit zentraler Aufhellung. Durch Konfluenz imponierte teilweise ein girlandenförmiges Bild (Abb. 1a–d). Dermatoskopisch stellten sich multiple, nicht wegdrückbare Petechien dar.

Differenzialdiagnostisch kamen sowohl multiple Erythemata migrantia bei Borrelieninfektion als auch eine Purpura anularis teleangiectodes und eine ungewöhnliche Vaskulitis in Betracht.

Auch eine Thrombozytopenie oder -pathie musste aufgrund der multiplen Petechien ausgeschlossen werden. Für eine hämorrhagische Kontaktdermatitis konnte kein auslösendes Agens eruiert werden, zudem fehlte hierfür die epidermale Beteiligung.

Histologisch imponierten zahlreiche, subepitheliale Erythrozytenextravasate und ein überwiegend lymphozytäres, perivaskuläres Infiltrat bei regelrecht stratifiziertem flach bis mittelbreitem Epithel.

**Figure 2:**
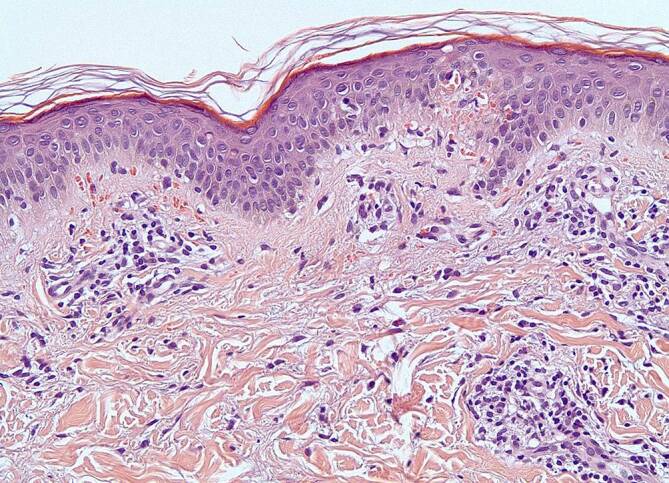

Es fanden sich weder fibrinoide Gefäßwandnekrosen noch eine Leukozytoklasie. In der Alcian-PAS(„periodic acid-Schiff reaction“)-Färbung zeigte sich kein Nachweis einer Mykose. In der Eisenfärbung konnten keine Eisenablagerungen ausgemacht werden, und die CD138-Färbung zeigte keine vermehrten Plasmazellen (Abb. 2).

## Diagnose

Angesichts der Histologie war eine hämorrhagisch pigmentierte Dermatose vorrangig in Betracht zu ziehen. Trotz der positiven IgM-Antikörper war eine Borreliose als Auslöser nicht wahrscheinlich, da sich weder die Histologie typisch für eine Borreliose zeigte noch ein kürzlicher Zeckenbiss erinnerlich war. Außerdem hatte die antibiotische Therapie mit Doxycyclin zu keiner Besserung des Hautbefundes geführt. Es ist daher von einer Seronarbe auszugehen.

In Zusammenschau von Histologie und klinischem Bild stellten wir die Diagnose einer Purpura anularis teleangiectodes.

## Therapie und Verlauf

Lokaltherapeutisch wurde Mometason 1 mg/g Creme angewendet.

Bei der Wiedervorstellung nach 20 Tagen zeigte sich der Hautbefund bereits deutlich gebessert (Abb. 1e–h). Lediglich ein dezentes, flächiges Erythem war verblieben.

## Diskussion

Die Purpura anularis teleangiectodes (PAT) wird als Sonderform der Purpura-pigmentosa-progressiva(PPP)-Gruppe betrachtet und wurde 1896 erstmals von Majocchi beschrieben [5].

Weitere Varianten der PPP, die in ihrer klassischen Form auch als Morbus Schamberg bezeichnet wird, sind die lichenoide Pigmentpurpura, die ekzematidartige Purpura und der Lichen aureus. Alle Unterformen weisen eine ähnliche Histologie mit lymphozytär dominiertem Entzündungsinfiltrat v. a. in der papillären Dermis und Erythrozytenextravasate auf [6].

Die Klinik und die Epidemiologie ermöglichen jedoch eine Differenzierung der Unterformen.

Die PAT zeichnet sich gegenüber den übrigen genannten hämorrhagisch pigmentierten Dermatosen durch ein anuläres Muster aus und findet sich vorzugsweise bei jungen Frauen.

In der Literatur finden sich nur wenige Berichte einer PAT. Die Tab. 1 zeigt eine Übersicht der publizierten Fälle.

**Table 1:** 

Autor Jahr	Geschlecht, Alter	Lokalisation	Vermuteter Auslöser	Histologie	Therapie	Verlauf
Spaulding [10] 2019	w, 63	Untere Extremität	Sklerotherapie	– Perivaskuläres, lymphozytäres Infiltrat – Erythrozytenextravasate – Hämosiderophagen in oberer Dermis	– Vortherapie mit Clotrimazol/Betamethasondipropionat-Creme nicht erfolgreich – Keine weitere Therapie	– Verblassen nach mehreren Monaten – Minimale residente Pigmentierung
Okhovat [6] 2017	m, 42	Arme, Beine, Rücken und Abdomen	Levofloxacin	– Interfacedermatitis – Oberflächlich perivaskuläres Entzündungsinfiltrat – Erythrozytenextravasate	– Vortherapie mit Econazol-Creme und Triamcinolonacetonid-Creme 0,5 % ohne Besserung – Levofloxacin abgesetzt	2 Wochen später abgeblasst, keine neuen Läsionen
Dhali [2] 2015	w, 12	Obere und untere Extremität	–	– Erythrozytenextravasate in Dermis und subkutanem Fett – Lymphozytäres Infiltrat in Dermis perivaskulär und periadnexiell	*Phototherapie:* – 3-mal wöchentlich PUVA, Start mit 1,8 J/cm^2^, 10 % Steigerung pro Sitzung, ab 10. Sitzung keine weitere Steigerung – Schmalband-UVB 3‑mal wöchentlich, Start mit 280 mJ/cm^2^ für 12 Wochen, 10 % Steigerung pro Sitzung – Anschließend 2‑mal wöchentlich für weitere 12 Wochen, ohne Steigerung (kumul. 16 J/cm^2^)	– Besserung ab 4,0 J/cm^2^ (10. Sitzung) PUVA – Nach 18. Sitzung PUVA: generalisierter Juckreiz, Übelkeit, Erbrechen, Bauchschmerz – Bei Verdacht auf PUVA-Intoleranz (kumul. 62 J/cm^2^) PUVA beendet – Unter Schmalband-UVB komplette Rückbildung
Kaplan [4] 2014	w, 30er	Untere Extremität, Gesäß	Isotretinoin	– Oberflächliche, perivaskuläre lymphozytäre Infiltrate – Erythrozytenextravasate – Hämosiderin in papillärer Dermis	Isotretinoin-Therapie beendet	– Abblassen der Läsionen nach 5 Wochen – Keine neuen Läsionen nach 3 Monaten
Wang [12] 2013	m, 85	Linkes Bein	Gentamicin, Quetiapin, Docusat Natrium, Nitrofurantoin und Hydralazin	– Perivaskuläres, lymphohistiozytäres Infiltrat – Erythrozytenextravasate	n.b.	n.b
Hoesly [3] 2009	w, 69	Untere Extremität	n.b.	– Perivaskuläre Lymphozyteninfiltrate – Erythrozytenextravasate	– Fluocinonid-Creme und Kompression – Pentoxifylline oral – Topische Calcineurininhibitoren Vitamin C – Triamcinolon-Injektionen intraläsional – Methotrexat oral 15 mg/Woche	– Vortherapien sine effectu – Triamcinolon-Injektionen: lokale Verbesserung, aber neue Läsionen – Komplette Abheilung nach 4 Wochen Methotrexat
Russell [7] 1950	w	Oberschenkel	n.b.	Erythrozytenextravasate	n.b.	– Nach 11 Monaten initiale Läsion fast komplett regredient – 4 neue anuläre Läsionen an den Oberschenkeln
Curle [1] 1930	w, 65	Rumpf, Gesäß, proximale Extremität	n.b.	n.b.	Totale Hysterektomie bei Uterusfibrom	Verblassen der Hautveränderungen 4 Wochen nach Operation
Semon [9] 1921	m	Untere Extremität	n.b.	n.b.	n.b.	n.b.

*m* männlich, *w* weiblich, *n.b.* nicht berichtet, *kumul.* kumulativ

In den veröffentlichten Fallberichten zeigt sich das typische klinische Bild anulärer Erytheme, die symmetrisch vorzugsweise an der unteren Extremität auftreten. Nur in einer Publikation wird ein Patient mit einem unilateralen Auftreten der Erytheme vorgestellt [12]. Über den Langzeitverlauf dieses Patienten wird jedoch nicht berichtet, sodass fraglich bleibt, ob dieser Patient nicht auch im Verlauf ein symmetrisches Auftreten entwickelte. Es wurden zuvor Fälle beschrieben, die zunächst unilateral begannen, jedoch letztlich alle symmetrische Läsionen entwickelten [7].

Die Ätiologie der PAT ist bisher ungeklärt. Während in den frühen Jahren nach der Erstbeschreibung ein Zusammenhang der Hautveränderungen mit einer kardialen Erkrankung vermutet wurde [9], ließ sich diese Annahme über die Jahrzehnte nicht bestätigen. Faktoren, die als Auslöser diskutiert werden, sind Medikamente und eine venöse Stauung. Es lässt sich mutmaßen, ob die initial berichteten Fälle der PAT in Zusammenhang mit kardialer Erkrankung nicht letztlich auf eine venöse Stauung kardialer Genese zurückzuführen sind.

Ein weiterer interessanter Fall, der diese Überlegung stützt, wurde von Curle und Smith 1930 beschrieben [1]. Es wird von einer 65-jährigen Patientin mit PAT berichtet, die zudem einen großen Abdominaltumor und klinisch eine Dilatation der oberflächlichen Venen aufwies. In diesem Fall könnte der Abdominaltumor zu einer venösen Abflussstauung geführt und somit als Auslöser der PAT gewirkt haben.

Die typischen Petechien und histologisch imponierenden Erythrozytenextravasate lassen differenzialdiagnostisch an eine Vaskulitis denken. Es fehlen jedoch regelhaft fibrinoide Gefäßwandnekrosen und eine Leukozytoklasie. Vielmehr kommt es durch die erhöhte Kapillarfragilität zum Austreten der Erythrozyten [11].

In den jüngeren Publikationen konnte zumeist ein auslösendes Agens gefunden werden.

Das Spektrum reicht von Antibiotika [2] über Retinoide [8] bis hin zu einer durch eine Sklerotherapie ausgelösten PAT [3]. In diesen Fällen führte ein Absetzen des auslösenden Medikaments zum progredienten Verblassen der Hautveränderungen [6]. In den als idiopathisch zu klassifizierenden Fällen führte mitunter – wie bei der von uns beschriebenen Patientin – bereits eine Lokaltherapie mit kortisonhaltigen Externa zu einer Besserung des Hautbefundes.

Die Rationale für die antientzündliche Therapie bezieht sich auf eine Aktivitätssteigerung des erworbenen Immunsystems [11].

Im Jahr 2015 wurde erstmalig eine Therapie der PAT mittels PUVA und Schmalband-UVB beschrieben [2]; 2009 beschrieben Hoesly et al. einen therapierefraktären Fall der PAT, der letztlich durch eine Therapie mit Methotrexat 15 mg innerhalb von 4 Wochen zu einer gänzlichen Abheilung kam [3].

Bezüglicher des häufiger auftretenden Morbus Schamberg konnten 2014 in einer retrospektiven Fallserie die Verträglichkeit und ein gutes Ansprechen der Hautveränderungen auf eine Kombination des Bioflavonoids Rutosid (2-mal 50 mg) mit Ascorbinsäure (1000 mg täglich) gezeigt werden. Der Wirkmechanismus beruht auf einer Reduktion freier Sauerstoffradikale, welche die vaskuläre Entzündung begünstigen [8]. Ähnliche Berichte für die seltenere PAT gibt es bisher noch nicht. Ein Therapieansprechen wäre im Sinne der ähnlichen Histologie mit gleichermaßen vorhandener kapillärer Fragilität jedoch auch bei der PAT gut denkbar.

Insgesamt stellt sich die Therapie der PAT schwierig dar, da auch die Ätiologie nicht vollständig geklärt ist. Eine antientzündliche Behandlung mittels topischer Kortikosteroide scheint auch hinsichtlich der lymphozytären Infiltrate vielversprechend. Bezüglich systemischer antientzündlicher Therapie liegen leider nur wenige Einzelberichte vor. Aufgrund der guten Datenlage der Anwendung von Rutosid und Ascorbinsäure beim Morbus Schamberg sollte diese Kombination auch bei der PAT erwogen werden. Jedoch fehlen zurzeit noch die Erkenntnisse, welche pathophysiologischen Unterschiede der Morbus Schamberg und die PAT besitzen, weswegen eine verlässliche Einschätzung der Übertragbarkeit des therapeutischen Ansprechens nicht möglich ist.

## Fazit für die Praxis

- Die Purpura anularis teleangiectodes ist eine seltene Variante der hämorrhagisch pigmentierten Dermatosen, die sich durch ihre anuläre Konfiguration der Erytheme und das vorwiegende Auftreten bei jungen Frauen auszeichnet.
- Als Auslöser können häufig Medikamente identifiziert werden, weswegen eine ausführliche Medikamentenanamnese unabdingbar ist.
- Bei idiopathischen Fällen ist oft eine Therapie mit kortisonhaltigen Externa und Kompressionstherapie erfolgreich.
